# Special delivery - extracellular vesicles released by commensal gut bacteria deliver bioactive protein to distal organs

**DOI:** 10.20517/evcna.2025.32

**Published:** 2025-11-19

**Authors:** Emily J. Jones, Aimee Parker, Rokas Juodeikis, L. Ashley Blackshaw, Arlaine Brion, Simon R. Carding

**Affiliations:** ^1^Food, Microbiome and Health Research Programme, Quadram Institute Bioscience, Norwich, NR4 7UQ, UK.; ^2^Core Science Resources, Quadram Institute Bioscience, Norwich, NR4 7UQ, UK.; ^3^Norwich Medical School, University of East Anglia, Norwich, NR4 7TJ, UK.; ^#^These authors contributed equally to this work.

**Keywords:** Bioengineering, BEV, Bacteroides, biodistribution, Nanoluciferase, microbe-host interactions, central nervous system

## Abstract

**Aim:** This study aims to investigate how the gut microbiota communicates with the host via bacterial extracellular vesicles (BEVs), given that direct contact between microbes and the healthy intestinal epithelium is prevented by a sterile mucin gel layer. Understanding these indirect interactions is critical because the specific pathways and mediators of microbiota-host interactions are incompletely understood. Tracking BEVs *in vivo* however is particularly challenging due to their nanoscale size and complex molecular composition.

**Methods:** To address these challenges, we developed a highly sensitive Nanoluciferase (NanoLuc) system for luminescence-based detection of BEVs produced by the model human commensal bacterium *Bacteroides thetaiotaomicron*. This approach was evaluated in germ-free and specific-pathogen-free mice, with comparisons between administration routes demonstrating the advantages of this system for *in vivo* BEV labelling over conventional lipophilic dyes.

**Results:** We report, for the first time, that BEVs endogenously produced in the gastrointestinal tract (GIT) of mice can deliver bioactive NanoLuc protein to multiple organ tissues, including the central nervous system. Our findings establish that naturally occurring BEVs in the GIT are capable of traversing multiple host barriers, including the intestinal epithelium, vascular endothelium, and the blood-brain-barrier, to access tissues such as the brain and eyes.

**Conclusion:** These findings advance our understanding of BEV-mediated microbe-host interactions and demonstrate the potential of BEVs as vehicles for long-distance delivery of bioactive therapeutics.

## INTRODUCTION

The intestinal microbiota is essential for maintaining human health, contributing significantly to digestion, nutrition, and immunity^[1]^. Increasing evidence shows that its influence extends beyond the gastrointestinal tract (GIT), affecting extra-intestinal organ systems, including the central nervous system (CNS) through bi-directional communication along the gut-brain axis^[2]^. However, the mechanisms by which specific microbiota constituents directly interact with host tissues and cells remain unclear, particularly in the lower GIT, where intestinal epithelial cells are protected by a sterile mucin gel layer that forms an impenetrable barrier to microbial contact^[3]^. Instead, interactions are more likely to occur via indirect mechanisms, including the release of secreted microbial products such as metabolites and bacterial extracellular vesicles (BEVs). These products can cross intestinal barriers, accessing underlying cells and the circulatory system^[4]^. BEVs, comprising DNA, RNA, and diverse bioactive compounds^[5]^, represent a particularly promising mode of microbe-host communication. This mechanism is supported by animal studies demonstrating BEV biodistribution^[6,7]^, and by the detection of GIT microbiota-derived BEVs in the bloodstream of both healthy individuals and patients with disease^[8]^.

A single microbial species can generate multiple BEV subtypes that vary in both size (40-400 nm) and protein cargo (proteome)^[5]^. *Bacteroides thetaiotaomicron* (*B. thetaiotaomicron*), a well-characterised human GIT commensal, releases BEVs with distinct proteomes, capable of modulating epithelial, immune, and neuronal cell function^[9,10]^. However, progress in this field is constrained by the technical challenge of visualising and tracking BEVs due to their nanoscale size. *In vivo* studies are further complicated by the physical similarities of BEVs to eukaryotic vesicles (such as exosomes) and the lack of specific bacterial reporters. Conventional approaches for BEV labelling typically involve non-covalent attachment of lipophilic fluorescent dyes. However, these dyes present several limitations. These include an extended half-life, alteration of particle size and membrane structure, promotion of vesicle aggregation, and formation of free-dye micelles similar in size to BEVs, all of which can obscure accurate biodistribution analyses^[11-14]^. Furthermore, such studies often involve parenteral or oral delivery of purified, pre-labelled BEVs, which does not reflect the dynamics of BEVs produced naturally within the GIT. It also remains unclear whether fluorescent dyes remain associated with BEVs during lysosomal degradation, potentially resulting in non-specific fluorescence signals in tissue imaging.

To address these challenges, the present study uses bioengineering to generate Nanoluciferase (NanoLuc)-labelled BEVs (NanoLuc-BEVs), enabling sensitive luminescence-based detection of vesicles generated by *B. thetaiotaomicron in vivo*. We demonstrate that BEVs naturally produced in the intestinal lumen can cross the epithelial barrier, enter the systemic circulation, and localise to immune-privileged organs such as the brain and eyes, delivering active NanoLuc protein. Furthermore, we evaluate how different labelling methods influence BEV tracing by comparing the *in vivo* biodistribution of NanoLuc-BEVs with that of lipophilic fluorescent 1,1’-dioctadecyl-3, 3, 3’, 3’-tetramethylindocarbocyanine perchlorate (DiD)-labelled BEVs.

## METHODS

### Bacterial strains and culture conditions

*B. thetaiotaomicron* was cultured in Brain Heart Infusion medium (BO1230B; Oxoid, UK) supplemented with 4 µM hemin (CAS16009-13-5; Merck, UK) or in Bacteroides Defined Medium (BDM4^[15]^) at 37 °C in an anaerobic cabinet. *Escherichia coli* (*E. coli*) DH10β was cultured in Luria-Bertani (LB; Oxoid) medium at 37 °C with shaking. Antibiotics (Merck, UK) were used at the following concentrations: erythromycin (20 µg/mL), gentamicin (200 µg/mL), and ampicillin (100 µg/mL).

### Molecular biology and cloning

A flexible plasmid vector system was developed for use in *B. thetaiotaomicron* (pBATH). In brief, the pGH044 shuttle plasmid was digested with restriction endonucleases *Eco53kI*/*SfoI* and re-ligated to excise a 331 bp fragment containing an *NdeI* restriction enzyme site. A Synthetic DNA fragment containing 5’ to 3’: *BamHI* restriction enzyme site; terminator BBa_B1001; BT_1830 promoter and ribosome binding site 7 (RBS7)^[16]^; protein coding sequence BT_3742 signal peptide (amino acids 1-32), tobacco etch virus (TEV) protease, mirRFP670nano near-infrared fluorescent protein, and two Strep-Tag II sequences; terminator BBa_B1007 and *PciI* restriction enzyme site (Supplementary Materials; Integrated DNA Technologies). This was inserted into the *BamHI*/*PciI* restriction enzyme site of the modified pGH044 vector, generating the pBATH.03 plasmid. For NanoLuc labelling, a synthetic fragment containing the coding sequence for NanoLuc replaced mirRFP670nano and the two Strep-Tag II sequences via insertion into the *XhoI/SpeI* restriction enzyme site of pBATH.03, generating the pBATH.51 plasmid (Supplementary Materials; Integrated DNA Technologies). A further pBATH.56 plasmid was generated by replacing the full coding sequence in pBATH.03 using the *NdeI/SpeI* restriction enzyme site. All cloning steps were performed in chemically competent *E. coli* DH10β cells.

Expression of NanoLuc was confirmed using the Nano-Glo Luciferase Assay System (N1110; Promega, UK) following the manufacturer’s guidelines, with 50 µL of sample and 50 µL of reagent combined. Luminescence was measured on a CLARIOstar Plate Reader (BMG Labtech, UK) using black, untreated polystyrene 96-well plates (Thermo Fisher Scientific, UK).

### Isolation and characterisation of BEVs

*B. thetaiotaomicron* cultures (500 mL) were centrifuged at 7,000 × *g* for 45 min at 4 °C to pellet bacterial cells, and supernatants were filtered through 0.22 µm pore-size polyethersulfone (PES) membrane filters (Sartorius, UK) to remove residual debris. The supernatants were concentrated by crossflow ultrafiltration (100 kDa molecular weight cut-off (MWCO), Vivaflow 50R, Sartorius), and the retentate was rinsed once with 500 mL of phosphate-buffered saline (PBS, pH 7.4). BEV suspensions (0.5 mL) were loaded onto a qEV/35 nm size-exclusion chromatography (SEC) column (Izon Science Ltd., France), and fractions were collected according to the manufacturer’s instructions. Fractions 1-4, containing isolated BEVs, were pooled and filtered through 0.22 µm pore-size PES membrane filters (Sartorius).

BEV hydrodynamic diameters and concentrations were determined by nanoparticle tracking analysis (NTA) using a ZetaView TWIN instrument (Particle Metrix, Germany) according to the manufacturer’s instructions. Analyses were performed with ZetaView software (version 8.05.12 SP1) using a two-cycle, 11-position, high frame rate protocol at 25 °C. Samples were diluted in ultrapure water to fall within the optimal detection range. Camera control settings: sensitivity 80; frame rate 30; shutter 100. Post-acquisition parameters: min brightness 20; max area 2,000; min area 5; trace length 30; 5 nm/class; 64 classes/decade.

### NanoLuc-BEV protease treatment

For TEV protease treatment, 90 µL of purified NanoLuc-BEVs (10^12^ particles/mL) were incubated with TEV protease (P8112; New England Biolabs, UK) under the manufacturer’s recommended conditions for 1 h at 30 °C. Fractions (0.5 mL) of treated and untreated NanoLuc-BEVs were collected using a qEVsingle 35 nm column (Izon Science Ltd.). For proteinase K treatment, 100 µL of purified NanoLuc-BEVs (10^11^ particles/mL) were incubated with proteinase K (P8107S; New England Biolabs) for 30 min at 37 °C according to the manufacturer’s guidelines. NanoLuc activity was measured as described above.

### *In vitro* assessment of NanoLuc-BEV cytotoxicity

The human colonic epithelial cell line Caco-2 (86010202; European Collection of Authenticated Cell Cultures, UK) was cultured at 37 °C in 5% CO_2_ in Eagle’s Minimal Essential Medium (EMEM) supplemented with 1% non-essential amino acids (M5650; Merck), 2 mM L-glutamine (G7513; Merck), 10% foetal bovine serum (F9665; Merck), and 1% penicillin-streptomycin (P4333; Merck).

NanoLuc-BEVs (10^10^/mL) were diluted in cell culture medium to final concentrations of 10^9^/mL (1:10) or 10^8^/mL (1:100) and applied to confluent Caco-2 monolayers cultured in collagen-coated (125-50; Merck) white, flat-bottom, 96-well plates (Nunclon delta-treated, Thermo Fisher Scientific) for 24 h. Cell viability was measured using the CellTiterGlo 2.0 reagent (G9242; Promega) according to the manufacturer’s instructions, with luminescence measured after 10 min on a CLARIOStar plate reader (BMG Labtech).

### Fluorescent DiD-BEV labelling

For DiD labelling, BEVs (10^11^/mL) were incubated with 5% (v/v) DiD Vybrant cell-labelling solution (V22887; Invitrogen Molecular Probes, UK) at 37 °C for 30 min. Unbound dye was removed by three washes with 0.5 mL PBS using centrifugal filters (100 kDa MWCO, Sartorius). DiD-labelled BEVs were further purified by SEC, checked for sterility, and characterised by NTA as above.

### Animal studies

Male and female specific-pathogen-free (SPF; C57BL/6) and germ-free (GF; C57BL/6) mice (8-12 weeks old) were housed in individually ventilated cages (SPF) or sterile isolators (GF) in adjacent rooms at the University of East Anglia Disease Modelling Unit. All mice were maintained under a 12 h light/dark cycle and received autoclaved water and either Rodent Maintenance 3 (RM3) diet (SPF) or RM3-Autoclavable diet (GF; Special Diets Services, UK). Drinking water was supplemented with vancomycin [50 mg/kg (1709007, Merck)], neomycin [100 mg/kg (N6386, Merck)], metronidazole [100 mg/kg (M1547, Merck)], amphotericin B [1mg/kg (PHR1662, Merck)], and ampicillin [1 mg/mL (A8351, Merck)] for three days, followed by a two-day washout. After monocolonisation, faecal pellets were collected at 3, 6, 14, and 21 days and snap frozen. All animal experiments complied fully with the Animal Scientific Procedures Act (1986), were approved by the UK Home Office, and received ethical clearance from the local Animal Welfare and Ethical Review Body.

### *In vivo* biodistribution imaging

To compare BEV labelling methods, GF mice received intravenous (IV) injections of DiD-labelled (*n* = 2), NanoLuc-labelled (*n* = 3), or dual-labelled DiD+NanoLuc-BEVs (*n* = 5) at a dose of 4 × 10^10^ particles, or PBS (200 µL/mouse). Animals were excluded from the study if full volume tail vein delivery was unsuccessful. Following injection, mice were housed in their original cage groupings to avoid cross-contamination of labelled BEVs via coprophagy. At 1 h post-administration, organs including eyes, brain, heart, lungs, liver, kidneys, spleen, mesenteric lymph nodes (MLNs), and thymus were excised. For monocolonisation, GF or SPF mice were administered a single oral gavage of 100 µL (OD600 = 0.72) of the relevant *B. thetaiotaomicron* strain or PBS. After 7, 10, 14, or 27 days of colonisation, Nano-Glo fluorofurimazine substrate (FFz, 0.44 µmol/mouse, CS320501; Promega) was delivered intraperitoneally 5 min before organ excision and whole blood collection. Blood was allowed to clot for 30 min at 20 °C, centrifuged at 1,500 × *g* for 10 min at 4 °C, and the serum was transferred into sterile 1.5 mL Eppendorf tubes for storage at -20 °C prior to Nano-Glo Luciferase Assay (Promega) as above.

*Ex vivo* images of organs were performed using an *In vivo* Xtreme multi-modal optical and X-ray small-animal imaging system (Bruker, UK) equipped with a back-illuminated 4 MP Charge-Coupled Device (CCD) detector with luminescence capability. Far-red DiD fluorescence (photons/s/mm^2^) was recorded using the following settings: excitation 650 nm, emission 700 nm, 19 cm field of view, 10 s exposure time, F-stop 1.1, and focal plane 0. Luminescence (Radiance: p/sec/cm^2^/sr) was recorded with no filter, 19 cm field of view, 30 s exposure, F-stop 1.1, and focal plane 0. Background images were captured by reflectance with 1 s exposure. Radiant efficiency for each organ was determined using Bruker Molecular Imaging software (v 7.2.0.21148). Image acquisition and analysis were performed blinded to treatment groups. Quantitative analysis was carried out in ImageJ/FIJI (version 1.52p; National Institutes of Health, USA) by overlaying foreground and background images, defining each organ as a region of interest (ROI), and recording total pixel counts (IntDen) as arbitrary units (AU). PBS controls were used to determine fluorescence and luminescence limits of detection (LOD).

### *Ex vivo* fluorescence imaging

Frozen brain and liver tissues from SPF mice intravenously administered DiD-BEVs (4 × 10^10^/mouse, 3 h) were embedded in optimal cutting temperature (OCT) compound (Agar Scientific, UK). Cryosections (6-8 µm) were prepared and nuclei stained with Hoechst 33342 (1:1,000, H10295; Thermo Fisher Scientific) for 30 min, followed by PBS washes and mounting using Fluoromount-G mounting medium (0100-01; SouthernBiotech, USA) and precision coverslips (Ibidi, Germany). Images were captured using a Zeiss LSM880 confocal microscope with a 63x/1.4 oil DIC objective and ZEN Black software (Zeiss, Germany). Fluorescence was recorded at 405 nm (blue; nucleus) and 647 nm (red; DiD). Channel intensities were adjusted for visualisation and standardised across treatment groups. Images were processed by thresholding, binarisation to remove background noise, and pseudo-colouring (yellow and cyan) using ImageJ/FIJI (version 1.52p; National Institutes of Health).

### *Ex vivo* tissue luminescence assay

Frozen organs, faecal pellets, and serum from GF mice monocolonised with *B. thetaiotaomicron* strains for 3, 6, 14, or 21 days were thawed and resuspended in PBS at the following concentrations: faecal pellets (100 mg/mL), spleen (500 mg/mL), liver (500 mg/mL), kidney (500 mg/mL), brain (1,000 mg/mL), and MLN (100 mg/mL). Samples were homogenised in lysing matrix D tubes (MP Biomedicals, USA) with protease inhibitor cocktail (S8820; Merck) using a FastPrep bead beater (MP Biomedicals), then centrifuged at 10,000 × *g* for 5 min at 4 °C. Supernatants and non-diluted serum (50 µL) were assessed for luminescence using the Nano-Glo Luciferase Assay (Promega), as above.

### Statistical analysis

Data are presented as individual values and mean ± standard deviation (SD) with sample sizes indicated. GraphPad Prism 5 (v5.04) was used for statistical analyses. Paired t-tests were applied for ZetaView analysis, and two-way analysis of variance (ANOVA) with Bonferroni post-tests used for dual-labelling and monocolonisation biodistribution studies. Differences between means were considered statistically significant at ^*^P ≤ 0.05.

## RESULTS

### Design of NanoLuc-containing *B. thetaiotaomicron* BEVs

To enable highly sensitive BEV detection, we incorporated NanoLuc^[17]^ into *B. thetaiotaomicron* BEVs. NanoLuc-labelled BEVs offer distinct advantages over previous methods for *in vivo* BEV detection^[18,19]^. NanoLuc is incorporated into the membrane of bioengineered BEVs that are naturally produced by the parent bacteria in the GIT, without requiring additional modification or labelling. The NanoLuc enzyme emits an extremely bright, stable signal, allowing for detection at much lower concentrations than fluorescent protein labels^[20]^. NanoLuc is also not affected by host tissue autofluorescence or photobleaching as it does not require excitation^[21]^. Unlike chemically derived fluorescent dyes, which can accumulate in host cells, NanoLuc is predicted to be degraded in host cell lysosomes together with BEVs^[22]^.

We designed a flexible plasmid system for *B. thetaiotaomicron* (pBATH) using a modified pGH044 vector backbone. The original coding region was replaced with a new promoter, ribosome binding site, and coding sequence flanked by two bidirectional terminators. This enables versatile genetic modification, such as N- and C-terminal tagging of the coding sequence.

**Figure 1:**
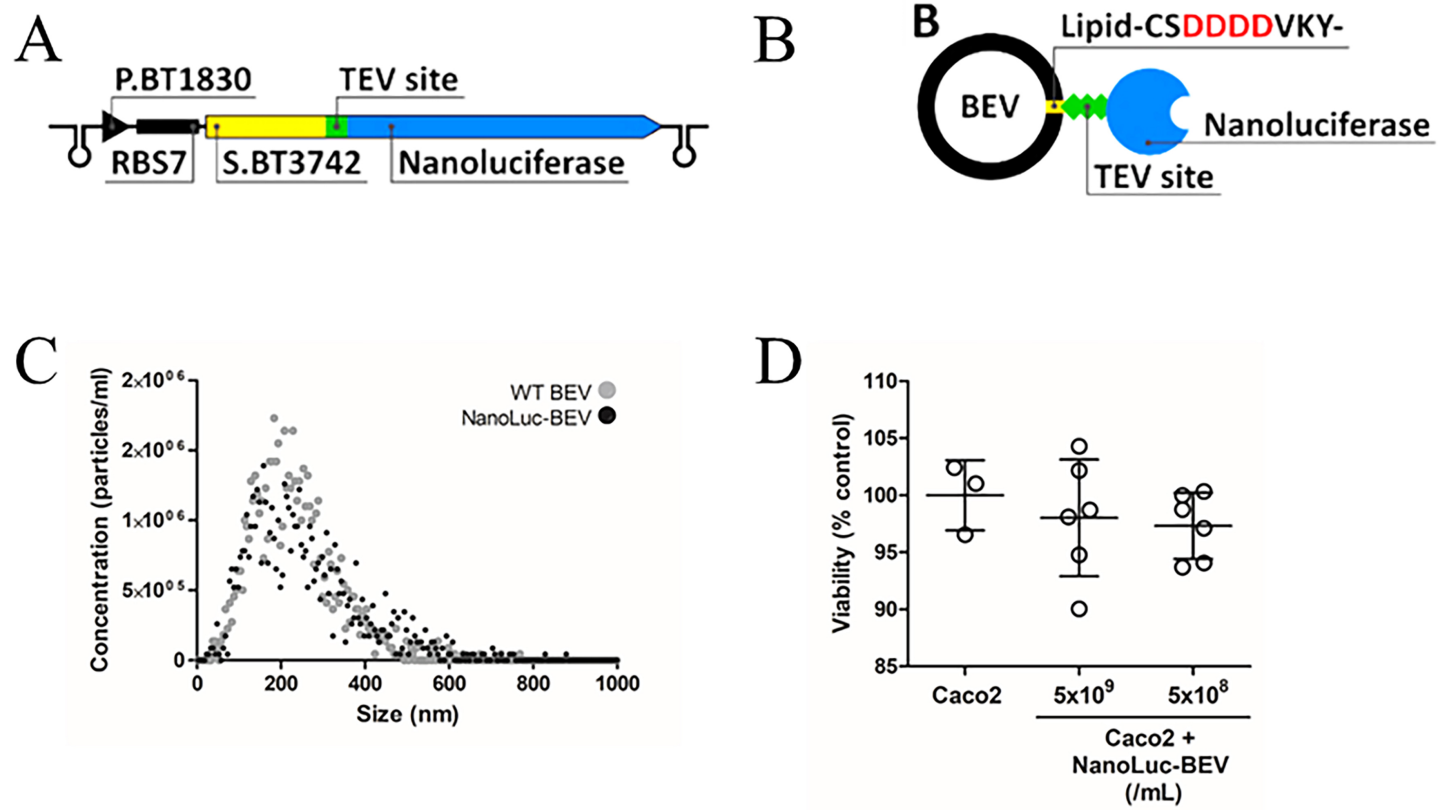
Design and characterisation of NanoLuc-BEVs. (A) BEV-targeted NanoLuc expression cassette cloned using the pBATH vector system. P.BT1830: promoter; RBS7: ribosome binding site; S.BT3742: signal peptide sequence; TEV site: TEV protease cleavage site. The cassette is flanked by bidirectional terminators; (B) Expected mature BEV-targeted NanoLuc structure. S.BT3742 directs the protein to the periplasm, where the signal peptide is cleaved and lipidated at Cys-24. The modified lipoprotein is inserted into the outer membrane, flipped to the outer leaflet, and incorporated into BEVs. Negatively charged aspartic acid residues upstream of the lipidation site facilitate this process (highlighted in the scheme); (C) Hydrodynamic diameter, distribution, and concentration of WT and NanoLuc-BEVs determined by nanoparticle tracking analysis; (D) Cytotoxicity of NanoLuc-BEVs to Caco-2 cells 24 h post-treatment, expressed as % viability relative to untreated control. Error bars represent mean ± SD. BEV: Bacterial extracellular vesicle; NanoLuc: Nanoluciferase; P.BT1830: promoter of BT1830; RBS7: ribosome binding site 7; S.BT3742: signal peptide of BT3742; TEV: tobacco etch virus; WT: wild type; SD: standard deviation.

Expression in *B. thetaiotaomicron* was driven by the BT_1830 promoter and RBS7, as defined by Whitaker *et al.*^[16]^. For targeted incorporation of NanoLuc into BEVs, the construct included an N-terminal lipoprotein signal peptide from BT_3742, followed by a TEV protease cleavage site and the NanoLuc coding sequence (pBATH.51; Figure 1A). The BT_3742 signal peptide features multiple negatively charged aspartic acid residues predicted to be lipidated [Figure 1B] and known to facilitate efficient incorporation of proteins into *B. thetaiotaomicron* BEVs^[23,24]^. A cytoplasmic NanoLuc-expressing control (pBATH.56) lacking this targeting sequence was also constructed to verify targeting specificity.

Verification of NanoLuc incorporation into BEVs was performed by assaying the culture supernatants of pBATH.51-expressing *B. thetaiotaomicron* cells. Approximately 75% of the NanoLuc signal was localised to the supernatant fraction during late stationary phase (Total: 40,991,369 ± 286,496 Relative Light Units; RLU; supernatant: 30,938,113 ± 141,499 RLU). To confirm surface display, isolated NanoLuc-BEVs were subjected to TEV protease cleavage, which cleaves the engineered site between the lipidated signal peptide and NanoLuc. Following size-exclusion fractionation to separate BEVs from cleaved protein, 80% of the NanoLuc signal eluted in the protein fraction, while 20% remained associated with BEVs, with no further loss observed after additional TEV treatment [Supplementary Figure 1]. This result may reflect limited accessibility of the cleavage site or a fraction of NanoLuc anchored to the inner face of the BEV membrane. To investigate this, NanoLuc-BEVs were treated with non-specific proteinase K, which resulted in a > 98% reduction in NanoLuc signal (non-treated = 1,142,667 RLU; treated = 20,783 RLU), confirming that the majority of NanoLuc is displayed on the surface of bioengineered BEVs. ZetaView measurements indicated no significant difference in mean hydrodynamic diameter between wild-type (266.25 ± 35.5 nm) and NanoLuc-labelled BEVs (251.8 ± 1.1 nm) indicating the engineering did not alter vesicle biophysical characteristics [Figure 1C].

### Impact of BEV labelling on *in vivo* biodistribution

Cytotoxicity assays in Caco-2 colonic epithelial cells demonstrated minimal impact on cell viability following treatment with NanoLuc-BEVs across all tested concentrations (5 × 10^9^/mL = 98.02%; 5 × 10^8^/mL = 97.33%; Figure 1D). These findings confirmed the suitability of NanoLuc-BEVs for biological administration.

**Figure 2:**
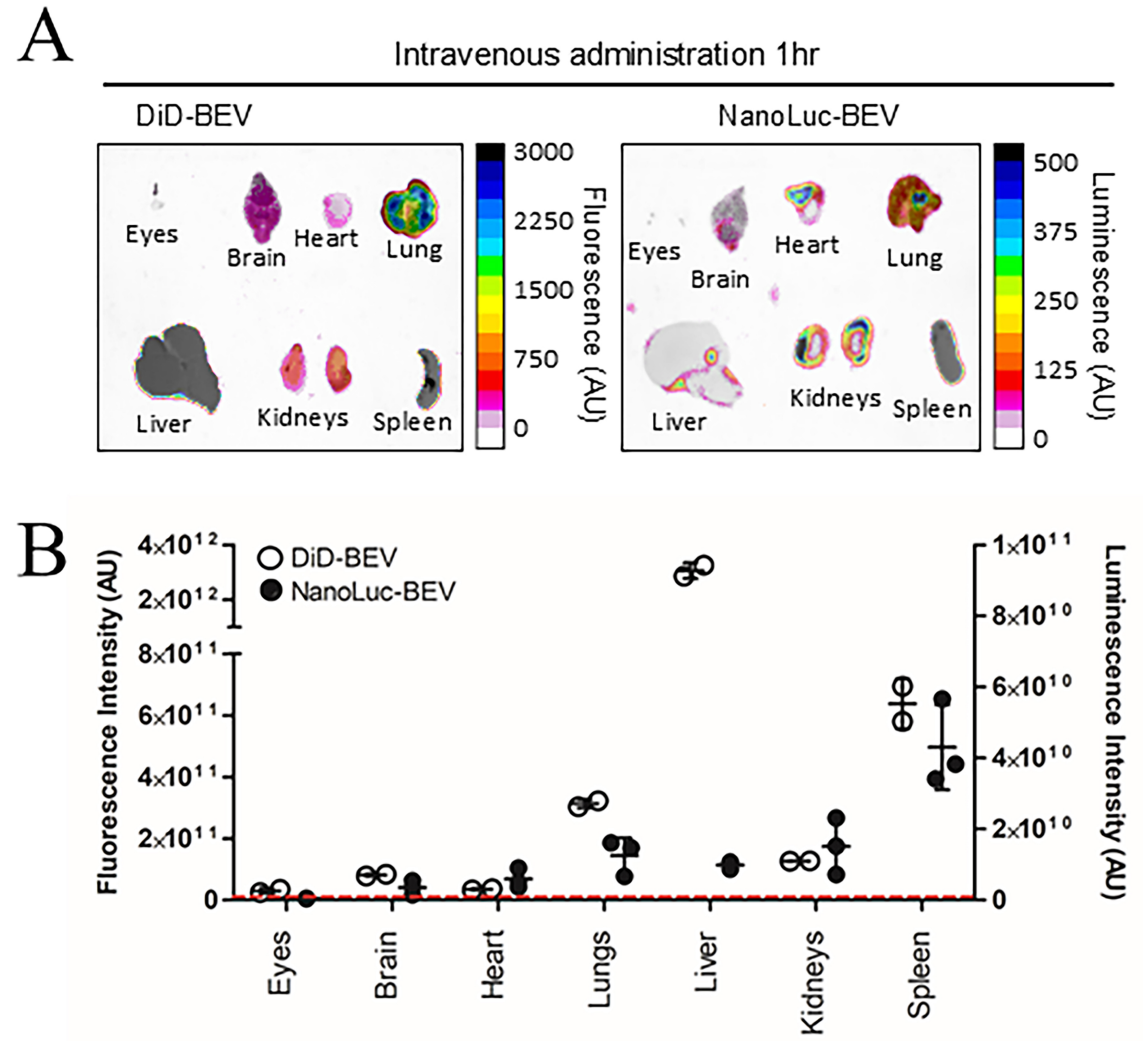
*In vivo* biodistribution of DiD- and NanoLuc-BEVs. (A) GF mice were intravenously administered DiD- or NanoLuc-BEVs (4 × 10^10^/mouse). Individual organs were excised 1 h post-administration and imaged using a Bruker *In vivo* Xtreme imaging system. The NanoGlo substrate was injected intraperitoneally 5 min prior to euthanasia and organ excision. Images represent *n* = 2 (DiD-BEV) or *n* = 3 (NanoLuc-BEV) mice per group; (B) Quantification of fluorescence (left axis) or luminescence (right axis) signals from each organ. Error bars represent mean ± SD. The red dotted line indicates the LOD. DiD: 1,1’-Dioctadecyl-3,3,3’,3’-tetramethylindodicarbocyanine; NanoLuc: Nanoluciferase; BEVs: bacterial extracellular vesicles; GF: germ-free; SD: standard deviation; LOD: limit of detection.

Subsequent *in vivo* experiments compared the biodistribution of NanoLuc-labelled BEVs and BEVs labelled with the lipophilic fluorescent dye DiD. Our previous studies showed systemic organ delivery following both oral and IV administration of DiD-labelled *B. thetaiotaomicron* BEVs, though IV delivery resulted in stronger organ signals^[9,25]^. Therefore, in the current study, IV administration of GF mice was used to deliver DiD-BEVs, NanoLuc-BEVs or dual-labelled BEVs to compare the specificity and sensitivity of labelling methods. After 1 h, both fluorescence and luminescence signals were quantified in excised tissues. The biodistribution pattern of DiD mirrored our previous findings, with the highest fluorescence signal observed in the liver, followed by the spleen, lungs, kidneys^[9]^, and brain^[25]^ [Figure 2A (left) and B (left axis)]. In contrast, NanoLuc-BEVs generated the highest luminescence in the spleen, followed by the kidneys, lungs, liver, heart, and brain [Figure 2A (right) and B (right axis)]. Notably, a detectable BEV signal was also observed in the eyes for both labelled BEVs [Figure 2A and B].

The single versus dual-labelled BEVs showed consistent DiD distributions, with no significant differences observed [Supplementary Figure 2A and B], while dual-labelling resulted in significantly increased NanoLuc signal in the lungs, indicating labelling may alter organ targeting (^*^P ≤ 0.05; Supplementary Figure 2C and D).

Building on this, further analysis revealed higher background fluorescence in tissues for DiD-labelled BEVs when compared to NanoLuc-BEVs [Supplementary Figure 2B and D], highlighting the increased sensitivity and reduced non-specific signal of NanoLuc-based tracing. Organ localisation differences between the two labelling methods were evident, with DiD signals favouring hepatic accumulation. To further visualise BEV localisation within excised organs, high-resolution confocal imaging of DiD-BEV tissues showed a diffuse liver fluorescence and a punctate peri-nuclear signal in the brain [Supplementary Figure 2E]. Due to reagent limitations, similar imaging of NanoLuc-BEVs could not be performed.

### GIT bacteria-derived BEVs enter host circulation for delivery of bioactive proteins

**Figure 3:**
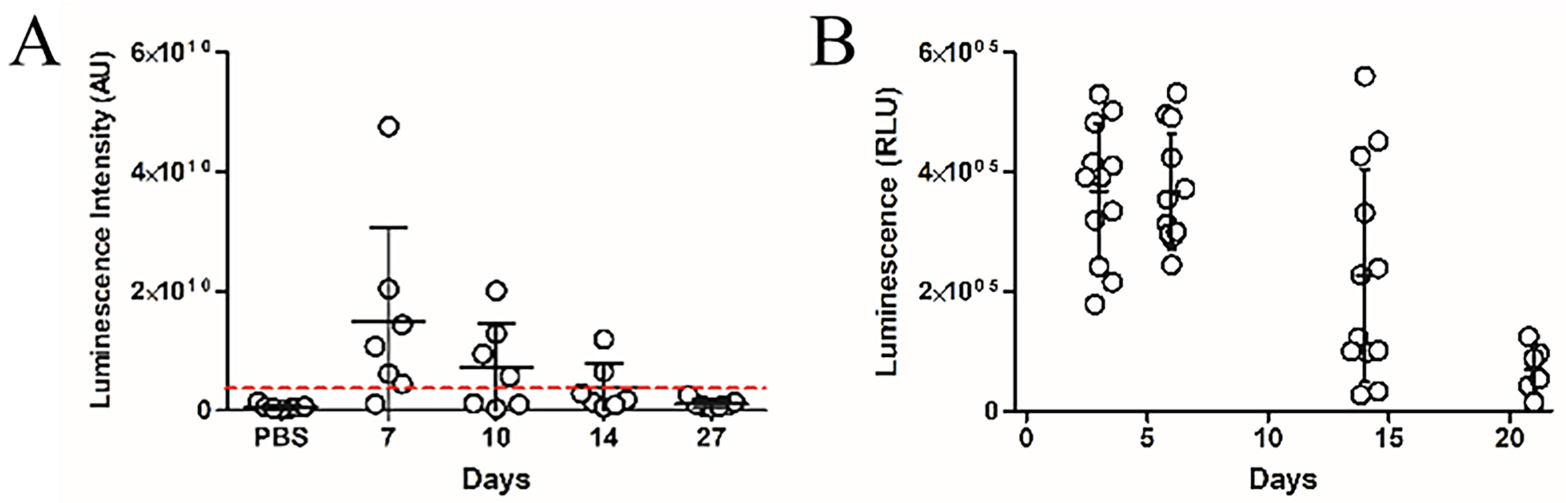
Time course of *in vivo* luminescence following colonisation with NanoLuc-BEV-producing *B. thetaiotaomicron*. GF mice were monocolonised at day 0*.* Samples were collected at 7, 10, 14, and 27 days post-colonisation. (A) Quantification of luminescence from organ tissues of animals administered PBS (*n* = 6) or the engineered *B. thetaiotaomicron* strain on day 7 (*n* = 6), 10 (*n* = 3), 14 (*n* = 2) and 27 (*n* = 4) post-colonisation. Each point represents the average luminescence for the eyes, brain, heart, lungs, liver, kidney, or spleen. Individual organs were excised for imaging using a Bruker *In vivo* Xtreme system. Substrate was injected intraperitoneally 5 min prior to organ excision; (B) Luminescence in the soluble fraction of faecal pellets from monocolonised GF mice. All samples were adjusted to 100 mg/mL prior to analysis (*n* = 12 for days 3, 6, and 14; *n* = 6 for day 21). Error bars represent mean ± SD. Red dotted line indicates LOD. NanoLuc-BEV: Nanoluciferase-bacterial extracellular vesicles; *B. thetaiotaomicron*: *Bacteroides thetaiotaomicron;* GF: germ-free; PBS: phosphate-buffered saline; SD: standard deviation; LOD: limit of detection.

Having established the capacity of IV administered BEVs to reach multiple organ tissues, we next evaluated whether BEVs naturally produced by colonising GIT bacteria could similarly enter the host circulation. GF mice were colonised with a *B. thetaiotaomicron* strain engineered to produce NanoLuc-labelled BEVs (pBATH.51). Organs including eyes, brain, heart, lungs, liver, kidney, and spleen were collected between 7 and 27 days post-colonisation and assessed for NanoLuc activity. Notably, luminescence was detected in all tissues, with a gradual decrease over time. The highest average signal occurred at 7 days post-inoculation and dropped below the LOD by day 27 [Figure 3A]. Luminescence in faecal pellet extracts also declined over 21 days [Figure 3B], which likely reflects the loss of the NanoLuc expression plasmid in the absence of antibiotic selection pressure and the metabolic burden of ongoing NanoLuc production. Minimal luminescence was observed in control animals receiving PBS or colonised with the cytoplasmic NanoLuc-expressing *B. thetaiotaomicron* strain (pBATH.56) [Supplementary Figure 3].

Having established the capacity of IV administered BEVs to reach multiple organ tissues, we next evaluated whether BEVs naturally produced by colonising GIT bacteria could similarly enter the host circulation. GF mice were colonised with a *B. thetaiotaomicron* strain engineered to produce NanoLuc-labelled BEVs (pBATH.51). Organs including eyes, brain, heart, lungs, liver, kidney, and spleen were collected between 7 and 27 days post-colonisation and assessed for NanoLuc activity. Notably, luminescence was detected in all tissues, with a gradual decrease over time. The highest average signal occurred at 7 days post-inoculation and dropped below the LOD by day 27 [Figure 3A]. Luminescence in faecal pellet extracts also declined over 21 days [Figure 3B], which likely reflects the loss of the NanoLuc expression plasmid in the absence of antibiotic selection pressure and the metabolic burden of ongoing NanoLuc production. Minimal luminescence was observed in control animals receiving PBS or colonised with the cytoplasmic NanoLuc-expressing *B. thetaiotaomicron* strain (pBATH.56) [Supplementary Figure 3].

**Figure 4:**
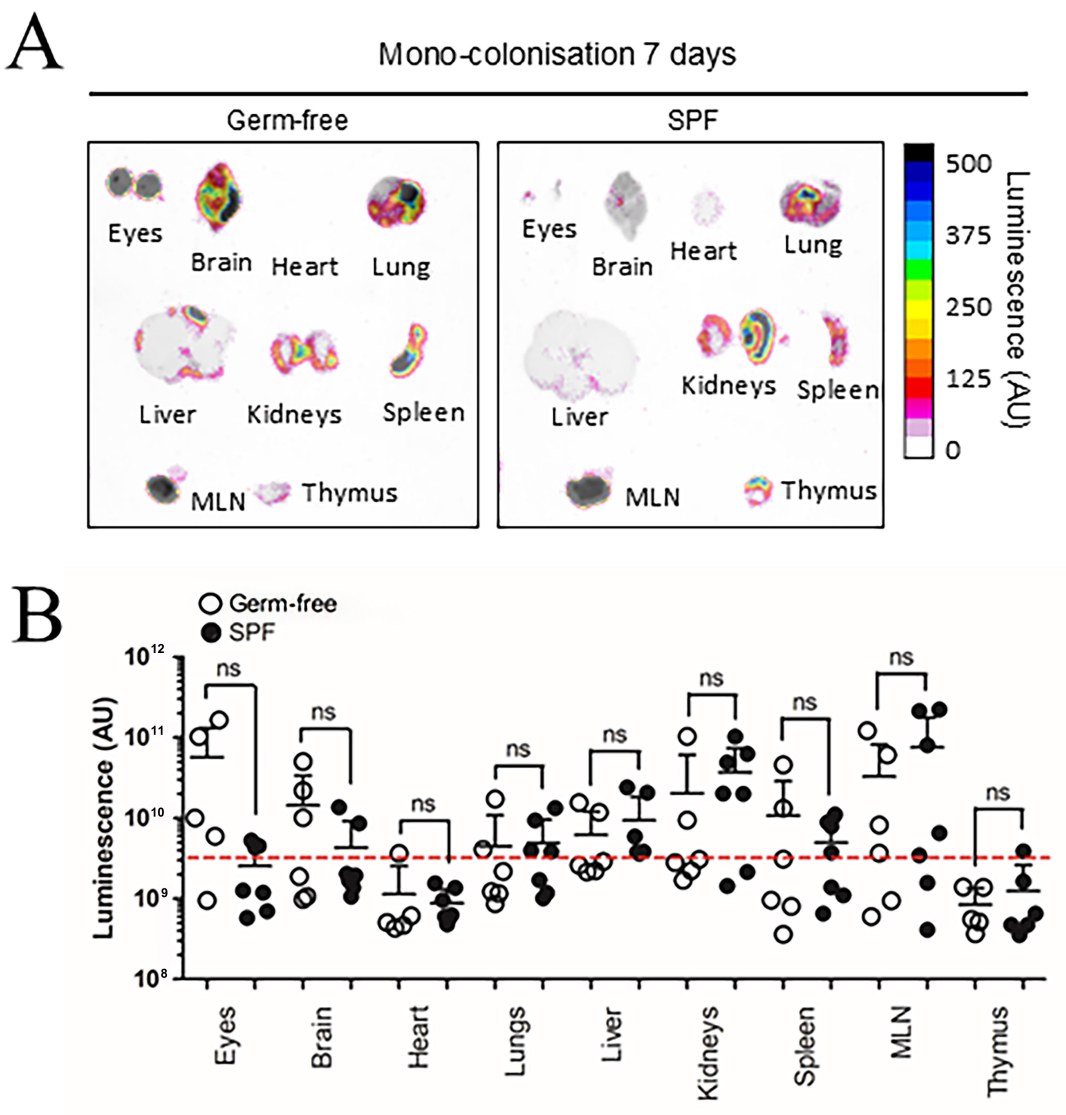
BEV distribution *in vivo* following colonisation with NanoLuc-BEV-producing *B. thetaiotaomicron*. (A) GF or SPF mice were monocolonised for 7 days, after which individual organs were excised and imaged using the Bruker *In vivo* Xtreme system. Substrate was injected intraperitoneally 5 min prior to euthanasia and organ excision. Representative images from GF (*n* = 6) or SPF (*n* = 7) mice are shown; (B) Quantification of luminescence from each organ. Error bars represent mean ± SD. Red dotted line indicates LOD. ns: Not significant; BEV: bacterially derived extracellular vesicle; NanoLuc: Nanoluciferase; *B. thetaiotaomicron*: *Bacteroides thetaiotaomicron*; GF: germ-free; SPF: specific pathogen-free; MLN: mesenteric lymph node; SD: standard deviation; ns: not significant; LOD: limit of detection.

**Figure 5:**
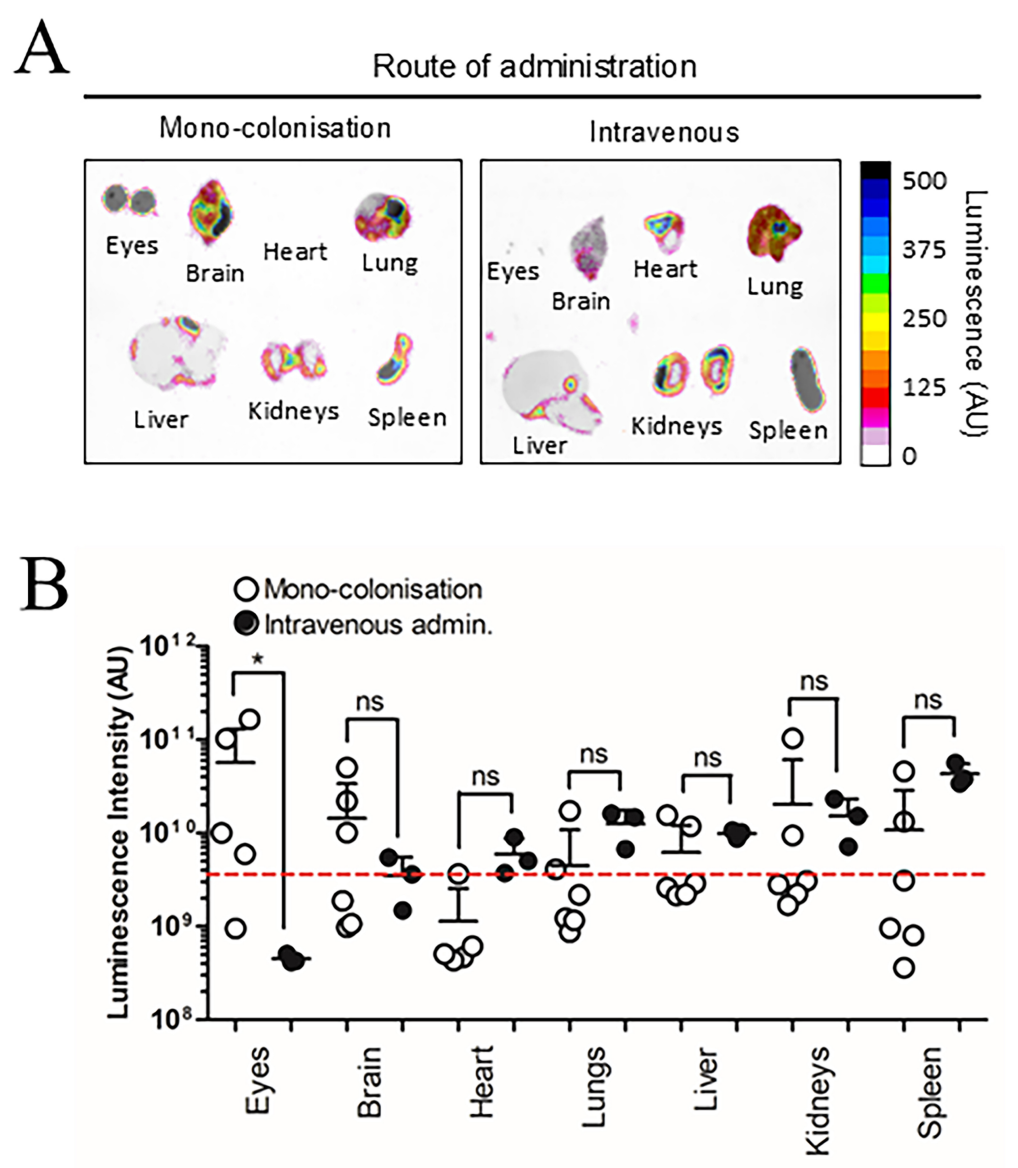
Route of administration alters NanoLuc-BEV biodistribution. GF mice were either monocolonised with NanoLuc-BEV-producing *B. thetaiotaomicron* or intravenously administered NanoLuc-BEVs (4 × 10^10^/mouse). (A) Organs were excised 7 days post-colonisation or 1 h post-IV administration and imaged using the Bruker *In vivo* Xtreme system. Substrate was injected intraperitoneally 5 min before euthanasia. Representative images from monocolonised (*n* = 6) or intravenously administered (*n* = 3) mice are shown; (B) Quantification of organ luminescence. Error bars represent mean ± SD. Red dotted line indicates LOD. ^*^P ≤ 0.05. ns: Not significant; NanoLuc-BEV: Nanoluciferase-bacterial extracellular vesicle; GF: germ-free; *B. thetaiotaomicron*: *Bacteroides thetaiotaomicron*; IV: intravenous; SD: standard deviation; LOD: limit of detection.

Previous research suggests BEV biodistribution in GF mice can be influenced by increased barrier permeability in intestinal epithelial cells and the blood-brain-barrier (BBB) due to reduced tight junction protein expression^[26,27]^. To control for these variables, SPF mice were evaluated alongside GF mice 7 days after colonisation with NanoLuc-BEV-producing *B. thetaiotaomicron*. Both SPF and GF groups displayed organ-level NanoLuc signals with no overall statistical differences, however, the highest luminescence signals were observed in the eyes and brains of some GF animals [Figure 4]. Compared to IV inoculation, monocolonised mice showed a significantly higher luminescence signal in the eyes (^*^P ≤ 0.05; Figure 5).

**Figure 6:**
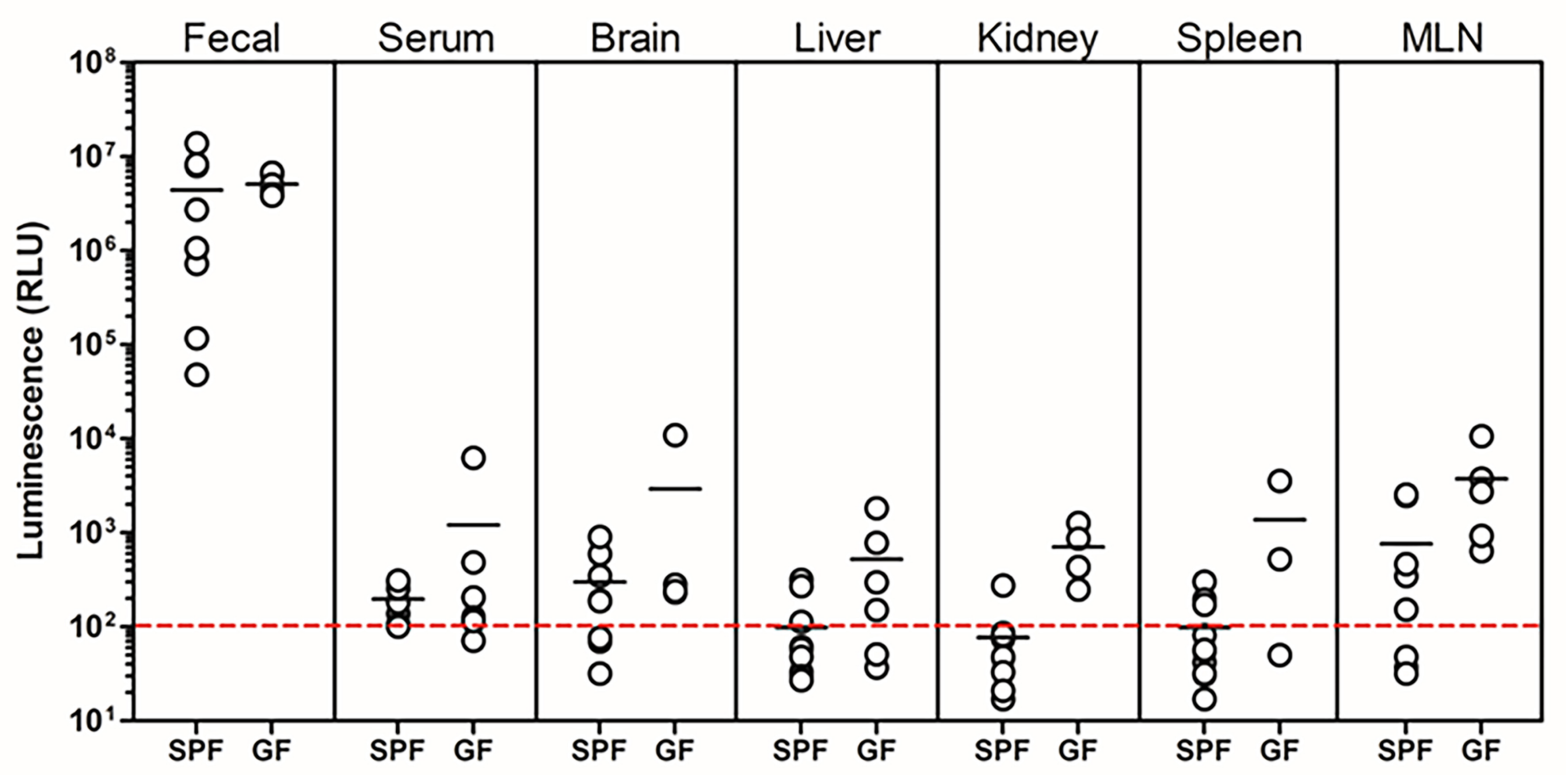
Organ distribution of NanoLuc activity following colonisation with NanoLuc-BEV-producing *B. thetaiotaomicron*. GF or SPF mice were monocolonised for 7 days. Individual organs were excised, snap-frozen, lysed, and luminescence quantified *in vitro*. Data represent 3-5 GF or 7-10 SPF mice per group. Error bars represent mean ± SD. NanoLuc: Nanoluciferase; BEV: bacterial extracellular vesicles; B. *thetaiotaomicron*: *Bacteroides thetaiotaomicron*; GF: germ-free; SPF: specific pathogen-free; SD: standard deviation.

To further confirm entry of NanoLuc-BEVs into the host circulation, soluble fractions from tissue homogenates and serum were assayed for luminescence. Following freeze-thawing, all tissue extracts displayed detectable NanoLuc activity, with GF mice showing the highest signals, in agreement with *ex vivo* whole-organ imaging results [Figure 6]. High variability was observed among samples, which we attribute to the freeze-thaw procedures required for sample preparation and transport prior to analysis.

## DISCUSSION

This study demonstrates that bioengineered NanoLuc-BEVs produced by the human commensal bacterium *B. thetaiotaomicron* can enter the systemic, hepatic, and lymphatic circulatory systems *in vivo.* They are capable of traversing multiple physical host barriers, including the intestinal epithelium, vascular endothelium, and BBB, and localise to various organ tissues, including those of the CNS. Critically, the NanoLuc protein remained catalytically active within organ tissues, highlighting the potential of BEVs as bioactive, long-distance therapeutic delivery vehicles within the host^[28]^. Prior studies of BEV trafficking primarily relied on administering pre-labelled BEVs via oral or IV routes, approaches that bypass the physiological dissemination mechanisms of BEVs that are naturally produced by the GIT microbiota.

The use of NanoLuc as a sensitive detection system has been well described for tracing mammalian extracellular vesicles (EVs) *in vivo*^[29]^. Here we utilised the technology to enable tracking of BEVs *in situ*, allowing direct comparison with fluorescent dyes. Lipophilic dyes, such as DiD, have long half-lives, which can lead to dye accumulation, non-specific signals, and altered nanoparticle biodistribution^[19,30]^. The advantages of NanoLuc include higher specificity, minimal background signal, reduced autofluorescence, and negligible photobleaching. Our findings show that DiD-labelled BEVs disproportionately accumulate in the liver, consistent with prior studies^[9,31]^. This is likely due to uptake by highly pinocytic liver sinusoidal endothelial cells (LSECs), where slow blood flow facilitates uptake^[32,33]^, or from trafficking of free DiD dye released after BEV degradation in host-cell lysosomes^[34,35]^. In contrast, NanoLuc labelling does not require membrane integration and is less likely to affect the natural tissue targeting of BEVs.

Our dual-labelling experiments revealed that BEV organ tropism is influenced by the choice of tracking method, highlighting that label selection can introduce experimental artefacts. While DiD-labelling casts light on common clearance pathways such as liver uptake, NanoLuc-labelling offers higher sensitivity and a greater signal-to-noise ratio. Nevertheless, NanoLuc-based tracking is not without limitations, including the requirement for luminescent FFz substrate diffusion in tissues, as well as rapid *in vivo* clearance, and increased NanoLuc signal in lungs and spleen^[22,36]^. Such challenges may reflect altered BEV surface protein composition due to bacterial bioengineering or degradation of NanoLuc during uptake and endo-lysosomal trafficking within host cells^[22]^. Overall, our findings emphasise that labelling strategies strongly influence *in vivo* biodistribution and underscore the need for careful interpretation of BEV trafficking data. To advance the use of BEVs as targeted therapeutic delivery vehicles, improved labelling systems will be essential to unravel organ-specific targeting mechanisms and quantify intracellular fate.

We observed that monocolonisation of mice with NanoLuc-BEV-producing *B. thetaiotaomicron* resulted in signals in the eyes, MLN, kidneys, brain, spleen, liver, lungs, heart, and thymus in both SPF and GF animals, the latter known to have more permeable intestinal and epithelial and blood-brain barriers^[27]^. To our knowledge, this is the first study to evaluate the biodistribution of endogenously produced, GIT microbiota-derived BEVs and to report their entry into the CNS *in vivo*.

Previous work, using pathogenic or pathobiont-derived labelled BEVs, has demonstrated that orally or intravenously administered vesicles access the brain via the vagus or trigeminal nerves^[37,38]^, or via circulation and crossing of the BBB^[39-42]^, potentially mediated by CNS monocytes/macrophages within the meninges^[43]^. Huang *et al.* also examined the biodistribution of bioengineered *E. coli* NanoLuc-BEVs, but these were delivered subcutaneously, and CNS trafficking was not evaluated^[44]^. Our study provides direct evidence that BEVs generated *in vivo* by the microbiota can also mediate CNS access. Supporting this, our brain imaging data and earlier *in vitro* gut-brain axis model findings show that *B. thetaiotaomicron* BEVs can traverse intestinal epithelial and BBB endothelial cell monolayers and be internalised by neuronal cells^[25]^. However, the precise pathways by which commensal BEVs transit from the gut to the brain *in vivo* remain to be clarified and will be vital for advancing their potential therapeutic application.

The biological consequences of commensal BEV uptake by neuronal cells require further investigation. Evidence from studies of pathogen-derived BEVs suggests that such interactions can contribute to cognitive impairment or behavioural changes in rodents^[45]^, potentially via neuronal damage^[39]^ or amyloid plaque accumulation and neurotoxicity^[40,46]^. By contrast, our *in vitro* gut-brain axis model indicates that commensal-derived *B. thetaiotaomicron* BEVs may instead drive protective anti-inflammatory and regulatory responses^[25]^.

Similarly, our observation of NanoLuc-BEV trafficking to the MLN suggests an immunomodulatory role, in line with their established function in filtering microbial products from intestinal lymph^[47]^, where transmigrating BEVs can interact with immune cells^[48,49]^. Indeed, 16S ribosomal RNA (rRNA)-based bacterial DNA signatures have been detected in MLNs of rats, likely in part derived from BEVs^[50]^. These findings provide further evidence that GIT microbiota-derived BEVs naturally disseminate through lymphatic pathways in hosts with intact intestinal barriers.

Although our study establishes proof-of-concept for NanoLuc as a reporter of BEV trafficking *in vivo*, several limitations should be acknowledged. Current *in vivo* imaging approaches do not resolve the cellular and subcellular distribution of BEVs in recipient tissues. Although confocal imaging confirmed punctate, perinuclear localisation consistent with previous *in vitro* models^[9,25]^, and diffuse hepatic distribution, higher-resolution imaging modalities and cell-type markers will be required to visualise BEVs at the single-vesicle level and to assess their intracellular fate. For clinical translation, understanding BEV internalisation, trafficking, degradation, and potential escape from endo-lysosomal compartments will be crucial to evaluate their utility for therapeutic cargo delivery.

Furthermore, the small study sample size, while sufficient for feasibility and consistent with the 3Rs (Replacement, Reduction and Refinement) principles, limits statistical power so future studies with larger cohorts will be needed. Additionally, the work here focused exclusively on BEVs from a single commensal species; it remains to be defined how BEVs from diverse microbial communities may alter host targeting, cargo delivery and functional impact.

Despite these limitations, this proof-of-concept study establishes the suitability of NanoLuc as a sensitive and physiologically relevant tool for tracking microbiota-derived BEVs in *vivo*, offering significant advantages over dye-based methods including higher sensitivity, lower background, and minimal disruption of natural targeting. Moving forward, further development of non-disruptive, high-sensitivity labelling techniques, together with expanded animal studies and nanoscale imaging, will be essential for fully realising the potential of BEVs as precision delivery vehicles for host-targeted therapies. Given the likelihood that BEVs may exploit neuronal or lymphatic routes, focused studies of these pathways will be essential for understanding their therapeutic potential.

In conclusion, this work provides the first direct evidence that BEVs endogenously produced by the gut commensal *B. thetaiotaomicron* have the capacity to access multiple organ systems and the CNS, including the brain and eye, when generated *in situ* within the GIT. These findings illuminate new routes of microbe-host communication and underscore the potential of BEVs as platforms for next-generation bioactive therapeutic delivery^[28]^.

## DECLARATIONS

### Availability of data and materials

The data that support the findings of this study are available from the corresponding author upon reasonable request.

### Copyright

© The Author(s) 2025.
