# Supplementary material for: Special delivery - extracellular vesicles released by commensal gut bacteria deliver bioactive protein to distal organs
Source: Extracell Vesicles Circ Nucl Acids. 2025 Nov 19;6(4):791–806. doi: 10.20517/evcna.2025.32 (PMC12809395; doi:10.20517/evcna.2025.32)
Supplement: Supplementary file 1 [file evcna-6-4-791-SupplementaryMaterials.pdf]

Supplementary Materials

Special delivery - extracellular vesicles released by commensal gut bacteria deliver bioactive protein to distal organs

Emily J. Jones[1,#], Aimee Parker[1,#], Rokas Juodeikis[1,#], L. Ashley Blackshaw[1], Arlaine Brion[2], Simon R. Carding[1,3]

[1]Food, Microbiome and Health Research Programme, Quadram Institute Bioscience, Norwich, NR4 7UQ, UK.
[2]Core Science Resources, Quadram Institute Bioscience, Norwich, NR4 7UQ, UK.
[3]Norwich Medical School, University of East Anglia, Norwich, NR4 7TJ, UK.
[#]These authors contributed equally to this work.

Correspondence to: Dr. Emily J. Jones, Food, Microbiome and Health Research Programme, Quadram Institute Bioscience, Norwich, NR4 7UQ, UK. E-mail: Emily.jones@quadram.ac.uk

Supplementary Information: Synthetic DNA sequences
>pBATH.03_DNA
GGACTCGTTAGCTACCTGAACATGTCTGACTTAAGAAAAAAAAACCCCGCTTCGGCGGGGTT
TTTTTTTGCATGCGTACATAGATCTCAAAAAGCGATGGATATGCCAAAACTTATTTTATTCATAT
AATCTATACAGTTCATCTTGGGGTTCGTTTACGAATATACGTTATTATAAGGAATAATCCGCTTC
TATTGTTTAAAAAGTGTGGAACAAATATGCTTTTTTGCCATTAATAACCTGTTGTCAGTCATTT
TGTCAGTCAATTTCTGCCAAACAATCATTATTTTGCTTTGGCATACTTTTCGCAACTGATTTAG
CGTCTTCTTCAAGAAGAGGACAAATCATGAATATAATGTATAACAAATAAAATCAAAAAAAAG
AACTGCTAGCGACTGATCGTAATCTTTAAAAAAAAATAAAACATATGAAAATAATGAAGAAAC
GTCTTTTGTACCTGTTCATGTTAATTTGCAGTATCTCCCTGTTTGTATCTTGTAGCGACGATGAC
GATGTGAAGTATGAAAATCTGTACTTCCAATCTCTCGAGATGGCTAATCTAGACAAGATGCTT
AACACTACTGTAACGGAAGTTCGGCAGTTTTTGCAAGTGGATAGAGTCTGTGTCTTCCAGTT
CGAGGAGGATTACAGTGGAGTGGTTGTGGTCGAGGCTGTTGATGATCGCTGGATCTCTATCTT
AAAGACCCAAGTACGCGATCGTTACTTCATGGAAACGAGGGGTGAAGAATATAGCCACGGCC
GATATCAGGCTATCGCGGACATTTATACAGCAAATTTAACCGAATGCTATCGTGACCTGTTGAC
ACAATTTCAAGTTCGTGCAATTCTTGCGGTGCCGATCCTCCAAGGGAAAAAACTGTGGGGTC
TGTTGGTAGCTCATCAGCTGGCAGCCCCTCGTCAATGGCAGACATGGGAAATAGACTTTCTG
AAACAGCAGGCCGTAGTAGTAGGAATTGCCATACAGCAGTCCCCATGGAGTGGCGGTGGAG
GATCTTGGTCACACCCACAGTTTGAAAAGGGTGGCGGGTCCGGAGGTGGTAGCGGAGGGTC
TTCGGCTTGGTCGCACCCGCAGTTCGAGAAATAAACTAGTCGCAAAAAACCCCGCCCCTGAC
AGGGCGGGGTTTTTTCGCGAATTCAGTCTAGGATCCGGTTATCATACGTACGCGA
>NanoLuc_DNA
GGACTCGTTAGCTACCTGACATATGCTCGAGGTGTTTACGCTGGAGGACTTTGTGGGGGATTG
GCGTCAGACAGCCGGGTACAATTTGGACCAAGTACTAGAACAGGGCGGTGTATCTAGCTTAT
TCCAAAATTTAGGCGTATCGGTAACCCCGATTCAGCGTATAGTACTGAGTGGCGAAAATGGCC
TTAAAATCGATATACATGTTATAATACCGTATGAAGGACTGTCGGGCGACCAAATGGGCCAGA
TCGAAAAAATATTCAAAGTCGTGTATCCAGTCGATGATCACCATTTCAAGGTTATCCTTCATTA
TGGTACACTTGTGATAGACGGAGTCACCCCCAATATGATTGACTACTTCGGTCGACCTTATGA
AGGTATTGCTGTTTTTGATGGTAAGAAAATTACGGTTACTGGAACATTGTGGAACGGAAATAA
GATCATCGATGAGCGTTTGATCAACCCTGATGGATCACTGCTGTTTAGAGTAACTATTAACGGT
GTTACTGGATGGCGCTTGTGTGAACGGATTTTAGCA

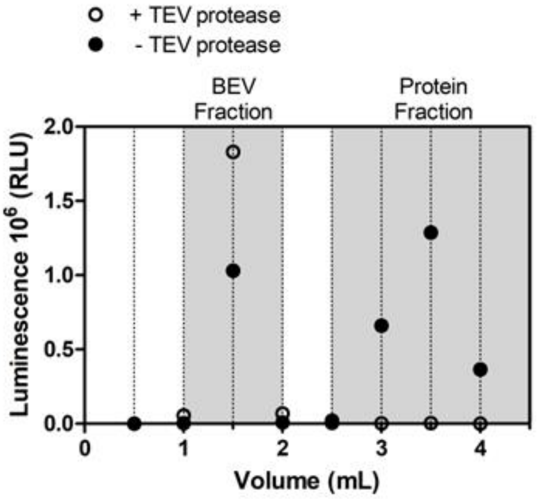

Supplementary Figure 1. NanoLuc is exposed on the surface of NanoLuc-BEVs. Isolated NanoLuc-BEVs were untreated or incubated with TEV protease for 1 h and 8 x 0.5 mL fractions collected for size exclusion fractionation and *in vitro* luminescence quantification. The grey areas depict the fractions containing BEVs or protein.

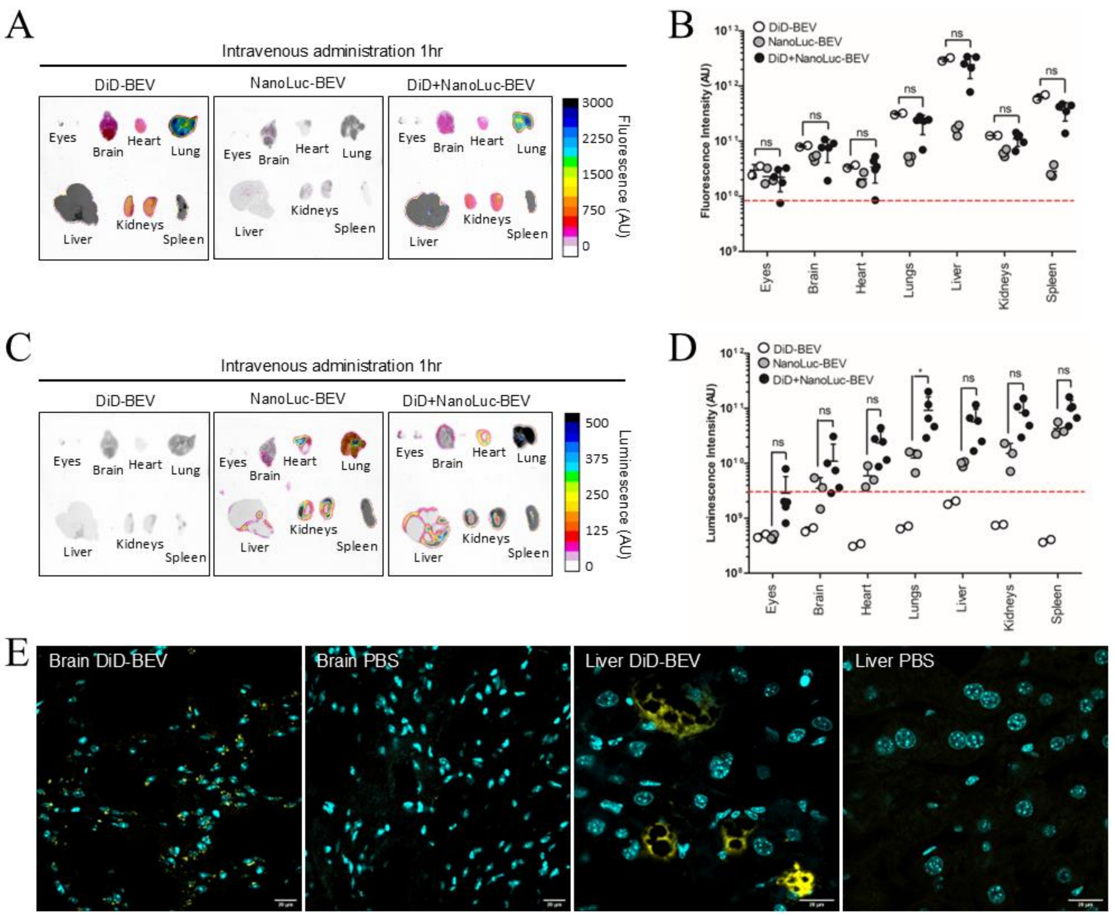

Supplementary Figure 2. *In vivo* biodistribution of DiD- and NanoLuc-BEVs. Germ-free mice were intravenously administered DiD-, NanoLuc- or dual labelled DiD+NanoLuc-BEVs (4 x 10^{10}/mouse). Individual organs were excised at 1 h post administration for imaging using a Bruker *in vivo* Xtreme imaging system. NanoGlo substrate was injected intraperitoneally 5 min prior to euthanasia and excision of individual organs. Quantification of the fluorescence (A-B) or luminescence (C-D) signal from each organ. Images are representative of those obtained from DiD-BEV (n=2; mice per group), NanoLuc-BEV (n=3) or DiD+NanoLuc-BEV (n=5). Error bars depict mean ± SD. * p = <0.05. ns = not significant. Red dotted lines depict LOD. Visualization of DiD-BEV biodistribution in brain and liver (E). SPF mice were intravenously administered DiD-BEVs (4 x 10^{10}/mouse). Individual organs were excised at 3 h post administration, snap frozen and tissues sectioned for staining. Images were captured using a Zeiss LSM880 confocal microscope and analysis performed in Image J/FIJI. Hoechst nuclear stain (cyan) and DiD-labelled BEVs (yellow). The images shown are representative of those obtained from PBS (n=2), or SPF (n=5) mice per group. Scale bars 20 µm.

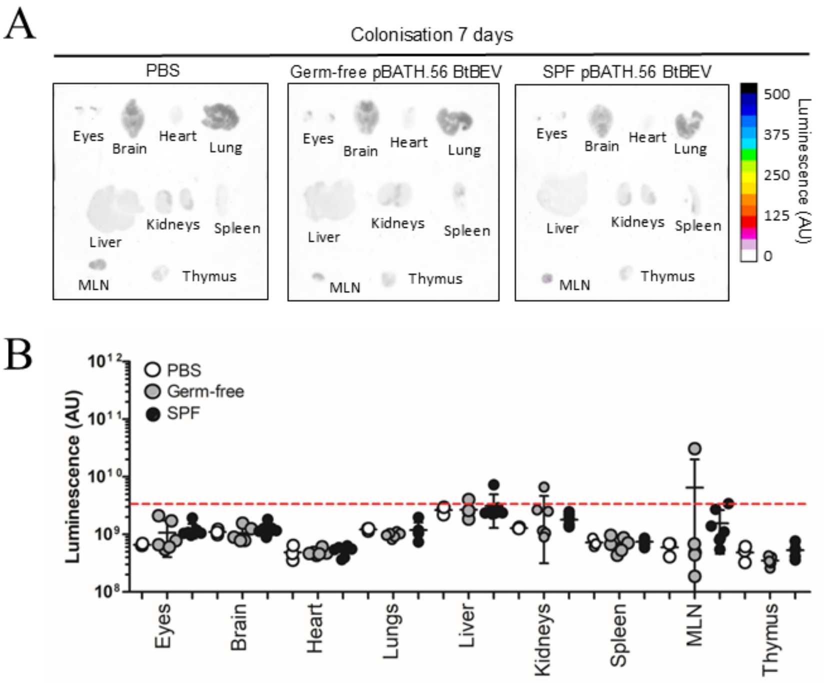

Supplementary Figure 3. *In vivo* Nanoluciferase signal distribution of controls. A. Germ-free or SPF mice mono-colonized with *B. thetaiotaomicron* producing cytoplasmic Nanoluciferase (pBATH.56) or PBS control. Individual organs were excised at 7 days post administration for imaging using a Bruker *in vivo* Xtreme imaging system. Substrate injection was performed intraperitoneally 5 min prior to excision of individual organs. The images shown are representative of those obtained from PBS (n=3), germ-free (n=6) or SPF (n=7) mice per group. MLN = mesenteric lymph nodes. B. Quantification of the luminescence signal from each organ. MLN = mesenteric lymph node. Error bars depict mean ± SD. Dotted line depicts LOD.
